# Fluoroestradiol (FES) and Fluorodeoxyglucose (FDG) PET imaging in patients with ER+, HER2-positive or HER2-negative metastatic breast cancer

**DOI:** 10.1186/s13058-025-01975-1

**Published:** 2025-02-17

**Authors:** Natasha B. Hunter, Lanell M. Peterson, Jennifer M. Specht, David A. Mankoff, Mark Muzi, Delphine L. Chen, William R. Gwin, Shaveta Vinayak, Nancy E. Davidson, Hannah M. Linden

**Affiliations:** 1 University of Washington, Seattle, WA USA; 2 Fred Hutchinson Cancer Center, Seattle, WA USA; 3 Universtity of Pennsylvania, Philadelphia, PA USA

**Keywords:** FES-PET, FDG-PET, HER2, Metastatic breast cancer

## Abstract

**Background:**

^18^F-Fluorodeoxyglucose (FDG) and ^18^F-Fluorestradiol (FES) have been FDA approved for measuring tumor glycolytic activity and estrogen receptor (ER) uptake, respectively, in clinical positron emission tomography (PET) imaging for patients with hormone-receptor (HR) positive metastatic breast cancer (MBC), but little is known about its utility in patients with breast tumors that overexpress human epidermal growth factor 2 (HER2). We hypothesize that comparing patterns of FDG and FES uptake in patients with HER2-positive versus HER2-negative MBC can guide further biologic and clinical studies into the HR/HER2-positive phenotype.

**Methods:**

We conducted a retrospective study examining uptake in matched lesions for FES and FDG-PET scans, assessing these parameters in 213 patients with ER-positive/HER2-positive (*n* = 33) versus ER-positive/HER2-negative MBC (*n* = 180). We employed log-rank and t-tests to assess the association of HER2 status with outcome variables and the hypotheses that patients expressing HER2-positive disease lived longer than patient with HER2-negative disease.

**Results:**

No difference in FES or FDG avidity was observed between patients with HER2-negative or HER2-positive tumor status. Limited data also suggests that patients with HER2-positive disease had better overall survival (*p* = 0.024), than those with HER2-negative disease, but not time-to-progression between the same patient cohorts.

**Conclusion:**

This retrospective analysis suggests that there is a possible role for future trials using FES-PET in helping to select patients with ER+/HER2-positive primary tumors who retain ER expression at all sites of disease and may benefit from endocrine therapy.

**Supplementary Information:**

The online version contains supplementary material available at 10.1186/s13058-025-01975-1.

## Background

^18^F-Fluorodeoxyglucose (FDG) is the most commonly used clinical PET tracer, for measuring tumor glycolytic activity. In 2020, the FDA approved ^18^F-Fluoroestradiol (FES) (*Cerianna*) as a PET imaging tracer for characterizing disease in patients with estrogen-receptor positive (ER+) breast cance [1]. FES-PET imaging has been shown in several studies, including key research by our group [2, 3, 4, 5], to accurately measure tumor ER expression at multiple tumor sites, which has been shown to predict response to endocrine therapy [6].

As FES-PET enters clinical practice, it is important to explore its utility in the full spectrum of patients with hormone (estrogen and/or progesterone) receptor positive breast cancer, including those with human epidermal growth factor 2 (HER2)-overexpressing tumors. Patients with HER2-overexpressing (HER2-positive) MBC are routinely treated with combination cytotoxic and HER2-directed therapy, given numerous trials establishing reduced mortality with addition of the HER2-targeted antibody trastuzumab to chemotherapy for this population [7]. While some older trials have demonstrated benefit from first-line combination of endocrine and HER2-directed agents [8, 9, 10], these regimens are currently used as initial therapy only for patients considered poor chemotherapy candidates, reflecting an understanding that providing chemotherapy plus HER2-directed therapy will offer more benefit than endocrine therapy for tumors overexpressing HER2.

A growing body of evidence points to dynamic interplay between estrogen and HER2 pathways, resulting in variable resistance or re-sensitization within these pathways [11, 12, 13]; this suggests a shifting spectrum of phenotypes that currently all fall under the general rubric of “triple-positive” disease [14, 15]. The recent emergence of a clinically relevant “HER2-low” phenotype supported by the efficacy of trastuzumab deruxtecan in patients with HER2 1 + and 2 + breast tumors [16] only emphasizes the need for a better understanding of HER2-driven disease and the crosstalk between these pathways.

Studies suggest that ER-positive, HER2-positive breast tumors may rely on both the endocrine and HER2 pathways for growth, and that patients with such tumors may derive less benefit from chemotherapy than those with ER-, HER2-positive breast cancer [14, 15, 17, 18] Pathologic investigations support these clinical observations. A Cancer Genome Atlas (TCGA) study found that only half of clinically HER2-positive breast tumors fell into a HER2-expressing genetic or epigenetic subtypes [19]. Other studies have demonstrated that HR-negative/HER2-positive tumors display higher nuclear and histologic grade, stronger HER2 immunohistochemistry (IHC) staining, higher average HER2/ chromosome enumeration probe 17 (CEP17) ratio, and greater p53 expression [20, 21] than their HR-positive/HER2-positive counterparts.

In the USA, our group has been investigating the potential of FES-PET imaging in characterizing ER + MBC, and has supported this technique for clinical approval and use. We conducted a retrospective study examining lesional uptake in matched contemporaneous FES and FDG-PET in patients with either ER-positive/HER2-positive or ER-positive/HER2-negative MBC. We hypothesize that comparing patterns of FDG and FES uptake in patients with HER2-positive versus HER2-negative MBC could provide insight into any differences between the two categories of ER-positive breast cancer and possibly help guide future clinical studies into this phenotype.

## Methods

### Patient selection

**Fig. 1:**
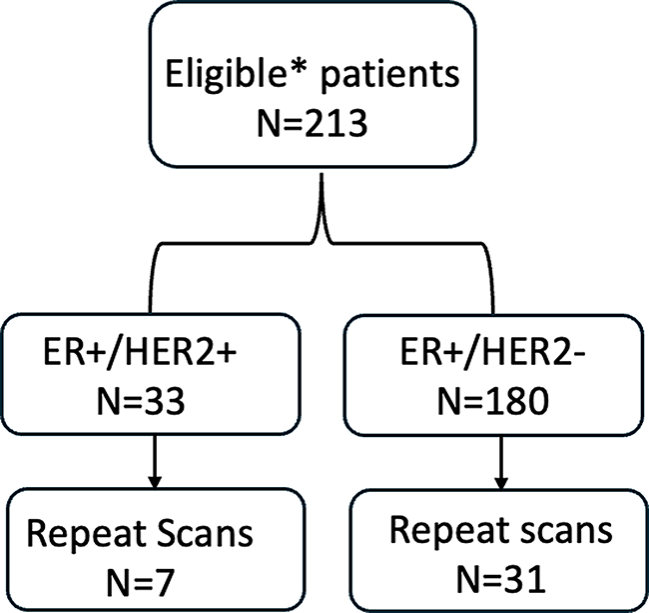
STARD diagram. *Eligibility included: ER + Primary. Any therapy except ER blocking. FES and FDG scans within 30 days without treatment change. Not currently enrolled in clinical trials

We retrospectively selected patients from our UW research FES PET database who had a history of biopsy-proven primary ER + breast cancer and who enrolled in one or more FES imaging trials. All patients had signed consent forms approved by the internal review board (IRB). Patients enrolled in on-going clinical trials were excluded. The STARD diagram in Fig. 1 illustrates how eligible patients were determined for this study. Subjects’ tumors were categorized as HER2-positive or HER2-negative according to contemporary ASCO/CAP guidelines [22]. Patients were included if they were either (1) Not on endocrine therapy, (2) on stable non-blocking endocrine therapy, or (3) at least 4 weeks off blocking therapy such as tamoxifen or fulvestrant. Chemotherapy was allowed at the time of FES imaging. Subjects were required to have FES and FDG-PET scans within 30 days of each other without an intervening changes in treatment. If patients had more than one scan that fit the eligibility criteria, only the first scan was used in the analysis, but the additional data is available in the supplemental data.

### PET imaging

Patients underwent both an FDG-PET and FES-PET study. PET imaging was performed by scanning multiple fields-of-view (FOV) of the torso from skull base to thighs on either a PET-only scanner (GE Advance) or PET/CT (GE Discovery STE) scanner. The scanners were cross-calibrated and monitored with a National Institute of Standards and Technology (NIST)-traceable reference source [23] to assure consistency of quantitative imaging measures [24].

FDG was prepared in-house or purchased commercially from Cardinal Health (Seattle, WA) and injected doses ranged from 260 to 407 MBq (median 350 MBq). All FDG-PET imaging was performed according to institutional clinical protocol as either a clinical or a research study.

FES was prepared and injected according to published methods and radiopharmaceutical quality specifcations [25, 26], and the dose ranged from 116 to 297 MBq (median 190 MBq). The FES-PET torso scan used the same imaging parameters as the FDG-PET scan. Fasting was required for FDG PET but not required for FES imaging.

### Image analysis

Images from the GE Advance PET scanner were reconstructed using 2D filtered back projection reconstruction (4.29 × 4.29 × 4.25 mm voxel resolution), while images from the Discovery PET/CT scanners used iterative 3D reconstruction (4.29 × 4.29 × 3.27 mm voxel resolution). All reconstructions had corrections for dead time, random events, scatter, sensitivity, decay, branching ration, and attenuation.

All patients had a positive FDG-PET scan, and only positive lesions on FDG-PET were used to determine FES status of the matched lesion. All lesions were defined clinically by CT or by PET/CT. We reviewed the maximum standardized uptake value (SUVmax) in up to 16 previously identified matched lesions between the two scans. Liver lesions were excluded due to physiological uptake of FES in the hepatic system. We then compared quantitative levels of tracer uptake in the scans for each lesion and its pair. FDG avidity was determined using standard clinical guidelines, typically lesions were defined as positive if they were visible above liver or aorta background. FES positivity was defined as any lesion with an SUVmax > 1.5, based on previous empirical experience [4, 27, 28]. If all lesions by FDG and FES were positive, the overall scan was read as positive; conversely if all lesions were FES negative, the scan was read as negative. An FES scan was called heterogeneous if at least one lesion with a corresponding FDG-avid lesion was below the FES threshold for positivity [2, 3, 25, 26].

### Clinical parameters

In addition to the clinical and pathological data used for study eligibility, we also recorded prior therapies including chemotherapy, endocrine therapy, and/or HER2-targeted therapy; therapy each patient was on at the time of the PET scans and what therapy they were on after the PET scans. Date of progression of disease (PD) was recorded for each patient; we estimated time-to-progression (TTP) as the interval between the FES-PET scan and progression by additional imaging (CT or FDG-PET) or by other clinical indications. Similarily, overall survival (OS) was recorded as the time from FES-PET scan to death.

### Statistical considerations

We employed a one-tailed, two-sample, equal variance (homoscedastic) t-test with the hypothesis that patients expressing HER2-positive disease lived longer than patient with HER2-negative disease.

To assess the association of HER2 status with the outcome variables TTP and OS, we created Kaplan-Meier plots and applied the log-rank test to determine probability using SPSS v23 statistical software (IBM Corp, worldwide). Although not part of the analysis, we also calculated whether FDG-PET or FES-PET was more predictive of outcome (see supplemental data).

## Results

### Patient population and scan results

Selection criteria matched 213 patients who were included for analysis in this retrospective study. Enrolled patients were imaged at the University of Washington between 1996 and 2018 with each scan occurring within 30 days of its corresponding match. The majority (74%) were postmenopausal, with a median age of 55. Of the 213 subjects, the tumors of 33 patients (15%) overexpressed HER2, consistent with population estimates of prevalence of HER2-positive disease [29]. 158 (74%) patients had ductal disease, and 35 (16%) had lobular disease. Of the 33 patients who had HER2-positive breast cancer, 28 (85%) had ductal carcinoma. There was similar distribution of patients with HER2-positive and HER2-negative disease having had prior endocrine therapy, with HER2-positive patients having had a higher percentage of prior chemotherapy (79% for HER2-positive, 63% for HER2-negative). There was also a similar distribution of patients on endocrine, chemotherapy or not on therapy at the time of the FES-PET scans, regardless of HER2 status. Approximately one third of the patients with HER2-positive disease were being treated with Herceptin (trastuzumab) at the time of their PET imaging. 30% (10/33) of the patients with HER2-positive disease went on chemotherapy after their FES-PET scan, which was slightly higher than the patients with HER2-negative disease (19%, 35/180). Details regarding demographic and pathological information are included in Table 1.

**Table 1:** Demographic, pathologic, and therapy data, *n* = 213 patients. Parameters include all patients and are also sub-divided by HER2 status

Patient/lesion characteristics	All patients, *n* = 213		
	Total	HER2-positive	HER2-negatve
**Age at FES**,** years (range)**	55 (23–79)	52 (28–74)	55 (23–79)
**Race**			
Caucasian	181 (85%)		
Asian	9 (4%)		
Black or African American	5 (2%)		
American Indian or Alaska Native	4 (2%)		
Unknown or Other	14 (7%)		
**HER2 status (n**,** % of total)**		33 (15%)	180 (85%)
**Menopausal status**			
Postmenopausal	158 (74%)	20 (61%)	138 (77%)
Premenopausal	50 (23%)	11 (33%)	39 (22%)
Male patients	5 (2%)	2 (6%)	3 (2%)
**Histology**			
Ductal	158 (74%)	28 (85%)	130 (72%)
Lobular	35 (16%)	4 (12%)	31 (17%)
Unknown	11 (5%)	1 (3%)	10 (5%)
Other	5 (2%)	0 (0%)	5 (3%)
Mixed	4 (2%)	0 (0%)	4 (2%)
Prior chemotherapy (n,%)	140 (66%)	26 (79%)	114 (63%)
Prior endocrine therapy (n,%)	109 (51%)	18 (55%)	91 (51%)
**Therapy at time of FES (n**,**%)**			
None	119 (56%)	16 (48%)	103 (57%)
Endocrine	80 (38%)	15 (45%)	65 (36%)
Chemotherapy	12 (6%)	2 (6%)	10 (5%)
Unknown	2 (1%)	0 (0%)	2 (1%)
Herceptin (HER2-positive only)		11 (33%)	
Chemotherapy after FES (n,%)	45 (21%)	10 (30%)	35 (19%)
Endocrine therapy after FES	142 (67%)	19 (57%)	123 (68%)
Unknown therapy after FES	13 (6%)	2 (6%)	11 (6%)
**FES scan machine**			
Advance	191	31	160
DSTE	22	2	20
**FDG scan machine**			
Advance	175	30	145
DSTE	35	1	34
FDG and FES done on same machine	185	29	156

### Imaging results

**Fig. 2:**
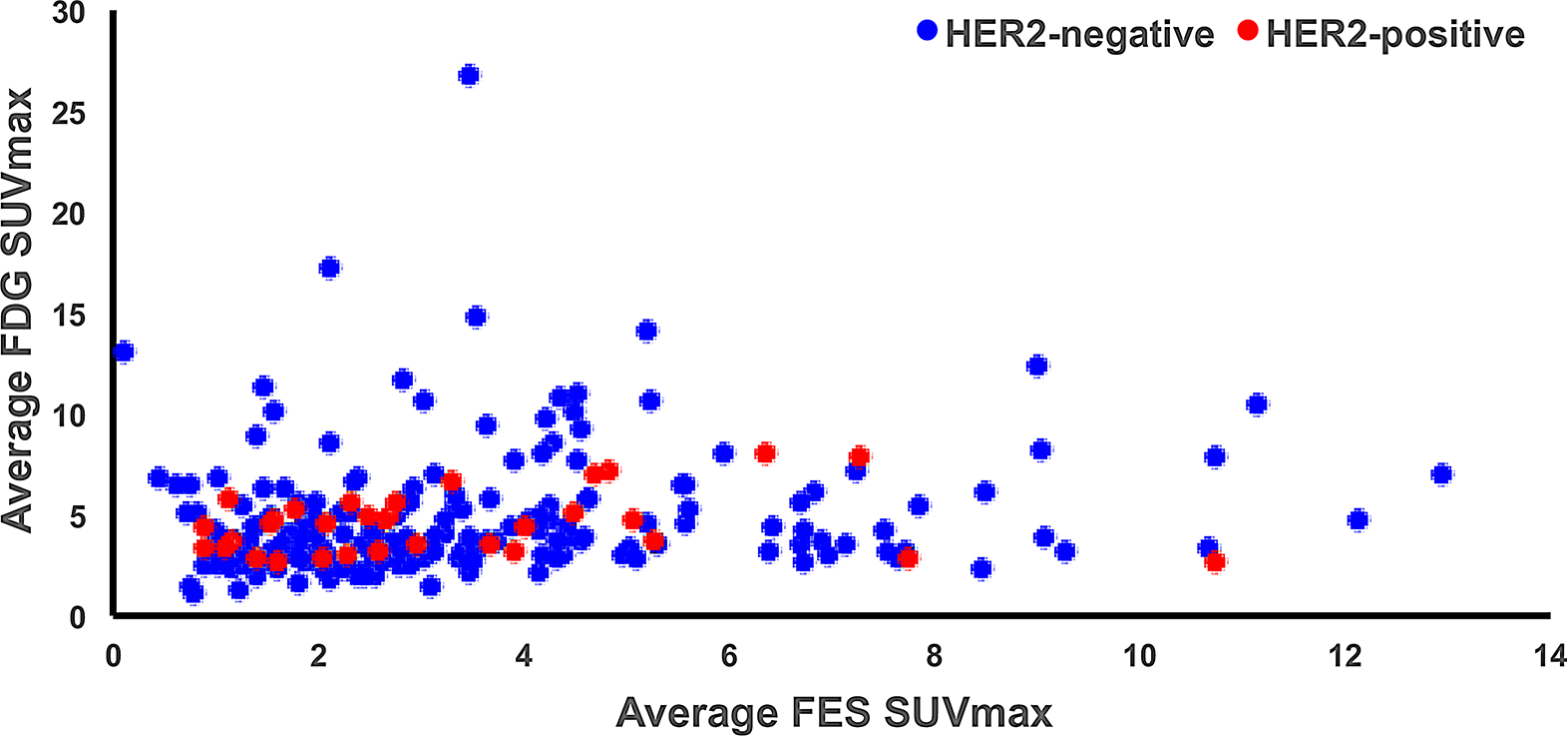
Average of FES SUVmax of all lesions across each patient against the average of FDG SUVmax for those same lesions for patients with HER2-positive (*n* = 33) and HER2-negative (*n* = 180) disease. High correlation was observed between FES and FDG average SUVmax across both phenotypes

A total of 1,039 metastatic sites were recorded (average = 5/scan (range 1,16)), with the majority (70%) representing bony lesions. The SUVmax of the lesions were averaged across all studies and across all patients to use in the data analysis. Table 2 details the FES and FDG uptake by lesion type and HER2 status. No significant difference in quantitative FES or FDG avidity was observed between soft tissue and osseous sites. Average FES and FDG SUVmax were similar among patients with either HER2-negative or HER2-positive tumor status across all studies and across all lesions (Table 2; Fig. 2). For patients with HER2-positive disease, the average FES SUVmax was 3.3 (0.88–10.4) across all studies and 3.6 (0.60–13.7) across all lesions. For patients with HER2-negative disease, the average FES SUVmax was 3.6 (0.12–12.9) across all studies and 4.2 (0.09–19.2) across all lesions.

The tumor heterogeneity, metastatic site (bone, soft tissue vs. visceral), and number of lesions were similar between HER2-positive and HER2-negative groups. A total of 73 FES scans (34%) were completely negative (*n* = 28, 13%) or had at least one FES negative lesion (*n* = 45, 21%), and 140 scans (66%) were considered completely FES positive. Of the 73 scans with completely negative or heterogenous scans, 14 (19%) had HER2-positive disease, compared with 19 (14%) with positive scans who had HER2-positive disease; i.e., there was no correlation between positive FES scans and HER2 status (Table 2).

**Table 2:** Imaging data, *n* = 1039 lesion in 213 scans. Parameters include all lesions in all scans and are also sub-divided by HER2 status

Patient/lesion characteristics:	Total *n* = 213 patients *n* = 1039 lesions	HER2-positive *n* = 33 patients *n* = 145 lesions	HER2-negative *n* = 180 patients *n* = 894 lesions
**Number of lesions per study: mean (range)**	5 (1–16)	4 (1–16)	5 (1–16)
**Lesion Distribution: n (%)**			
Bone	733 (71%)	100 (69%)	633 (71%)
Soft tissue/lung	306 (29%)	45 (31%)	261 (29%)
**FES average SUVmax overall: mean (range)**			
Average FES SUVmax across all studies	3.6 (0.12–12.9)	3.3 (0.88–10.4)	3.6 (0.12–12.9)
Average FES SUVmax across all lesions	4.1 (0.09–19.2)	3.6 (0.60–13.7)	4.2 (0.09–19.2)
**FES average SUVmax by tissue type**			
Average across all studies			
Bone	3.9 (0.46–11.2)	3.7 (1.0–11.0)	3.9 (0.46–11.2)
Soft tissue/lung	3.2 (0.11–13.4)	2.9 (0.09–9.9)	3.3 (0.12-13.0)
Across across all lesions			
Bone	4.4 (0.36–18.8)	3.7 (0.60–13.7)	4.5 (0.36–18.8)
Soft tissue/lung	3.4 (0.09–19.2)	3.3 (0.7–11.0)	3.4 (0.09–19.2)
**FDG average SUVmax overall**			
FDG SUVmax across all studies	4.9 (1.2–26.7)	5.3 (1.9–11.6)	5.0 (1.1–26.7)
FDG SUVmax across all lesions	5.1 (1.1–26.7)	4.4 (1.5–11.6)	5.2 (1.1–26.7)
**FDG average SUVmax by tissue type**			
Average across all studies			
Bone	5.0 (1.4–17.3)	4.9 (2.6-8.0)	5.0 (1.4–17.3)
Soft tissue/lung	4.8 (1.1–26.7)	3.9 (1.8–5.9)	5.0 (1.1–26.7)
Across across all lesions			
Bone	5.2 (1.4–25.0)	4.7 (1.9–11.6)	5.3 (1.4–25.0)
Soft tissue/lung	4.8 (1.1–26.7)	3.8 (1.5–9.6)	5.0 (1.1–26.7)
**FES qualitative interpretation**			
FES positive (no lesions < 1.5 SUVmax)	140 (66%)	19 (58%)	121 (67%)
Average FDG uptake			
Average FES uptake			
FES negative (no lesions > 1.5 SUVmax)	28 (13%)	4 (12%)	24 (13%)
Average FDG uptake			
Average FES uptake			
FES heterogeneous (at least one lesion < 1.5 SUVmax)	45 (21%)	10 (30%)	35 (19%)
Average FDG uptake			
Average FES uptake			

### Progressions and survival

**Fig. 3:**
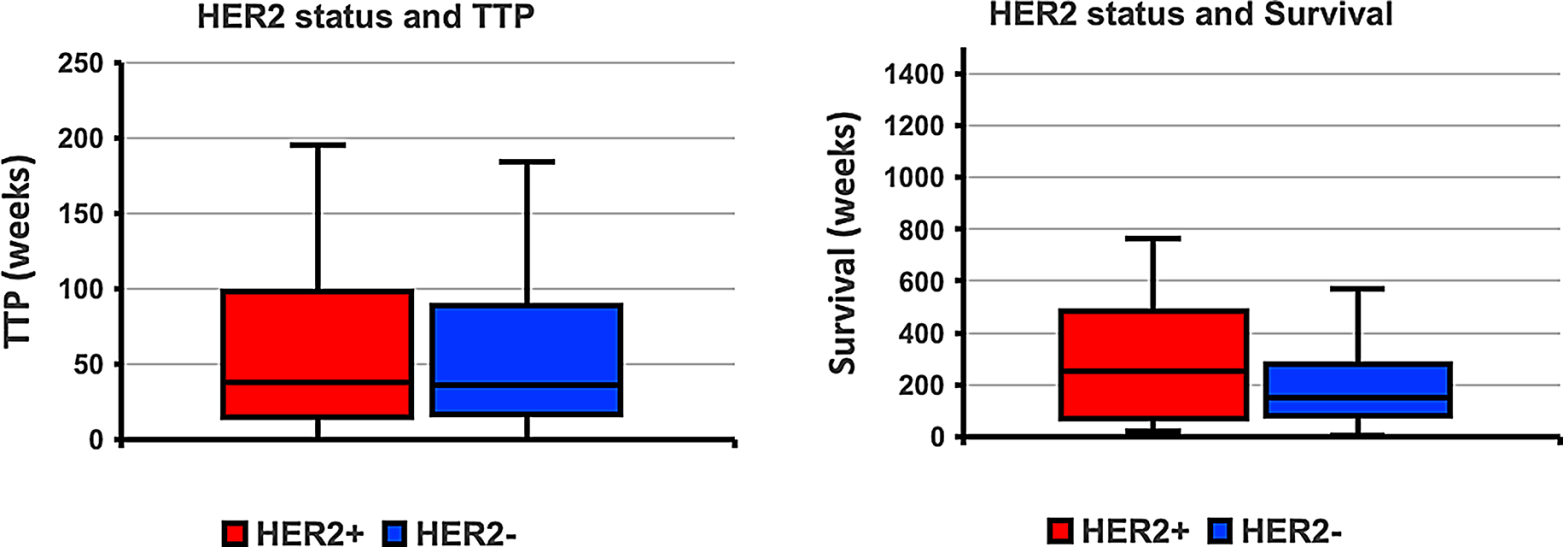
Comparison of TTP and OS for documented events in patients with HER2-negative disease and those with HER2-positive disease showed that patients with HER2-positive disease lived longer than those with HER2-negative disease (*p* = 0.024) while there was not a significant relationship for HER2 status with TTP (*p* = 0.152)

**Fig. 4:**
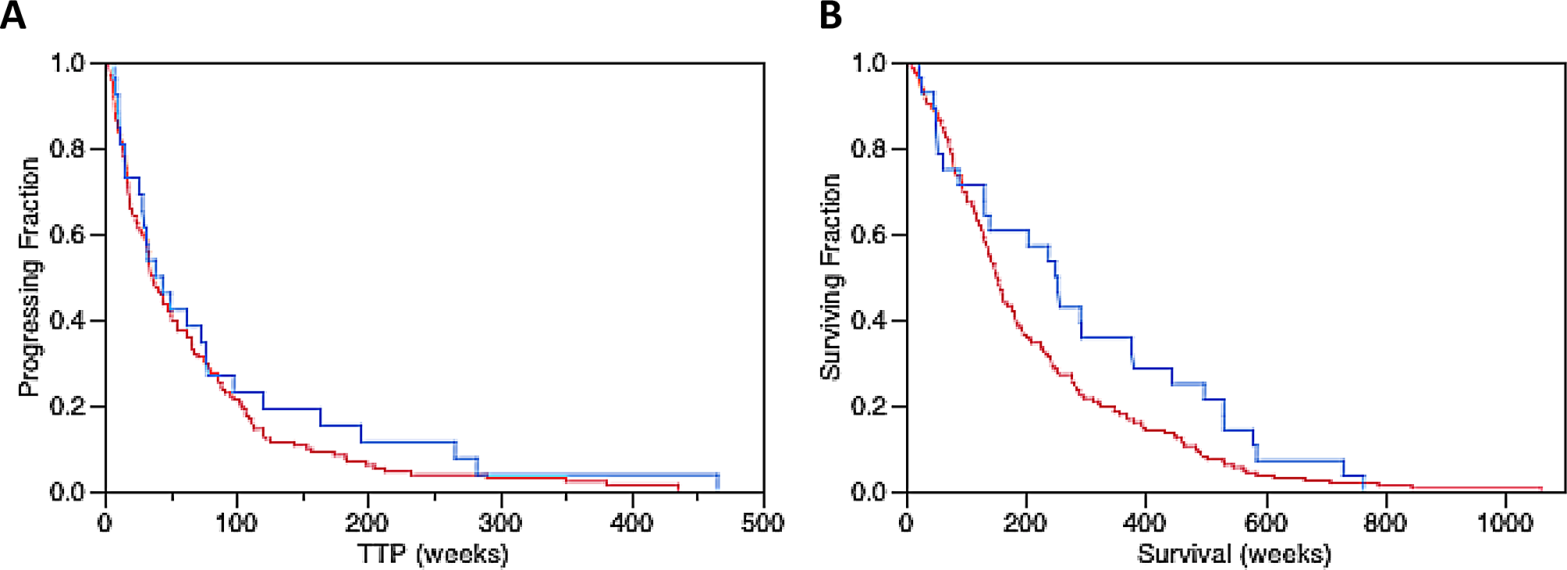
Kaplan-Meier outcome analysis examining the association between HER2 status and time-to-progression (TTP) (**A**) and overall survival (OS) (**B**). The red line is patients with HER2- disease, while the blue line is patients with HER2 + disease. TTP had 157 patients in the analysis with 131 with HER2-negative disease and 26 with HER2-positive disease. OS analysis had 190 patients with 162 having HER2-negative disease and 28 with HER2-positive disease. Log-rank tests showed that for HER2 status the relationship was not significant for TTP (*p* = 0.323) or for overall survival (*p* = 0.087)

Table 3 notes TTP and OS by HER2 status measured from the time of the FES-PET scan. Mean TTP in the HER2-positive subset was 86.3 (range 7.3-465.8) weeks, compared to the HER2-negative subgroup of 66.8 (range 0.7-436.1) weeks. There was no significant difference between these groups with respect to TTP (*p* = 0.152). OS in the HER2-positive subset had a mean of 286.1 (range 22.0-1480.4) weeks vs. 211.2 (range 3.0-1060.7) weeks in the HER2-negative subgroup. Applying a one-tailed, two-sample, equal variance (homoscedastic) t-test comparing these two groups, showed that patients expressing HER2 + disease lived longer than patients with HER2- disease (*p* = 0.024) (Fig. 3). Additional Kaplan-Meier outcome analysis examined the association between HER2 status and TTP and between HER2 status and OS. These log-rank tests showed that for HER2 status the relationship was not significant for TTP (*p* = 0.323) or OS (*p* = 0.087) (Fig. 4). Supplemental data Table S1 shows the comparison of median FES and FDG uptake as it relates to TTP and OS.

**Table 3:** Time-to-progression and overall survival calculated from time of the FES-PET scan for patients with documented follow-up. 131 of the 180 (73%) patients with HER2-negative disease had documented time-to-progression events and 162 (90%) had recorded survival data. For the 33 patients with HER2-positive disease, 26 (79%) had documented progression and 28 (85%) had survival data

	Total *n* = 213	HER2-positive *n* = 33	HER2-negative *n* = 180
Weeks to progression: Mean (range)	70.1 (0.7-465.6)	86.3 (7.3-465.6)	66.8 (0.7-436.1)
Weeks to death: Mean (range)	222.2 (3.0-1060.7)	286.1 (22.0-764.7)	211.2 (3.0-1060.7)

### Additional data

In a separate sub-set analysis, thirty-eight (38) patients in the original cohort had 2 or more FES/FDG paired scans. These serial scans allowed tracking FES uptake over multiple timepoints. In the 38 patients who had serial scans, FES expression generally remained constant despite the fact that many underwent treatment during the interim (see supplemental data).

## Discussion

FES-PET has multiple potential clinical applications in the clinical practice of breast medical oncology aside from its proven utility in measuring estrogen receptor activity in patients with HR positive MBC and in predicting response to endocrine therapy.

We saw no difference in the rate of positive FES scans or average SUVmax uptake between patients with HER2-positive and HER2-negative disease, which we found notable given the common conception that HER2 rather than endocrine signaling is the dominant growth pathway in patients with HER2-positive disease. This result is intriguing, but potentially limited by selection bias of our historic data base of patients on various imaging sudies. Further investigation into the molecular genomic characteristics of these tumors is also warranted to determine if they may reflect previously described HER2-negative vs. luminal-type HER2-positive disease, and if so, to explore a combined radiologic plus pathologic biomarker approach.

The results of this retrospective analysis suggest that more research is indicated to investigate the hypothesis that presence or absence of FES-PET may be useful in discriminating between a hormonally active HER2-positive tumor that could respond to a non-chemotherapy treatment backbone, versus a tumor in which the HER2 pathway is dominant and thus less likely to respond to a chemotherapy-free, endocrine+/HER2-targeted approach. If borne out, our findings could support use of FES-PET as a selection tool to risk-stratify patients for enrollment to clinical trials.

Limitations of this trial include the lack of treatment stratification for subjects and the cross-sectional nature of the database, the scarcity of available metastatic biopsies, as well as the type of patients referred for FES imaging trials. Liver lesions are not well characterized by FES-PET, as the tracer is hepatically cleared and the liver thus appears strongly positive by FES-PET. This limits analysis of patients with liver lesions and many of the referred patients with ER+/HER2-positive tumors were bone or soft tissue dominant which is associated with more indolent disease course across breast cancer subtypes [30]. This is a retrospective study dating back several years with imaging done on two different machines over the length of time encompassing the data collection. It would be interesting to investigate the degree of ER positivity in patients with HER2-positive versus HER2-negative tumors, as lower levels of ER activity have been suggested to correlate with a more aggressive phenotype and low response to endocrine therapy [31]. Unfortunately, standard pathologic assessment of ER and PR receptor status varied across this time frame, limiting our ability to compare receptor expression level. PR status was also not documented in all cases. Another limitation relates similarly to the retrospective nature of the trial, as treatment algorithms and regimens have also varied across the two-decade time span involved.

## Conclusions

In a cohort of ER+, HER2-positive and HER2-negative patients undergoing concurrent FDG and FES-PET scans, FES and FDG uptake were similar in both HER-negative and HER2-positive MBC, as was the fraction of lesions with absent FES uptake suggesting ER expression loss. These observations suggest a possible role for FES-PET in helping to select patients with ER+/HER2-positive primary tumors who retain ER expression at all sites of disease that may benefit from endocrine therapy in future trials of treatment options for this patient group.

## Electronic supplementary material

Below is the link to the electronic supplementary material.

Supplementary Material 1

## Data Availability

No datasets were generated or analysed during the current study.

## Abbreviations

FDG: ^18^F-Fluorodeoxyglucose
FES: ^18^F-Fluorestradiol
PET: Positron Emission Tomography
FDA: Federal Drug Administration
ER: Estrogen Receptor
HR: Hormone Receptor
MBC: Metastatic Breast Cancer
HER2: Human Epidermal Growth Factor 2
IHC: Immunohistochemistry
CEP17: Chromosome Enumeration Probe 17
IRB: Internal Review Board
FOV: Fields-of-view
NIST: National Institute of Standards and Technology
SUVmax: Maximum Standardized Uptake Value
PD: Progression of Disease
TTP: Time-to-progression
OS: Overall Survival
