# Supplementary material for: Fluoroestradiol (FES) and Fluorodeoxyglucose (FDG) PET imaging in patients with ER+, HER2-positive or HER2-negative metastatic breast cancer
Source: Breast Cancer Res. 2025 Feb 17;27:23. doi: 10.1186/s13058-025-01975-1 (PMC11834562; doi:10.1186/s13058-025-01975-1)

Supplemental Data:

Fluoroestradiol (FES) and Fluorodeoxyglucose (FDG) PET imaging in patients with ER+, HER2-positive or HER2-negative metastatic breast cancer

Natasha B Hunter, Lanell M Peterson, Jennifer M Specht, David A Mankoff, Mark Muzi, Delphine L Chen, William R Gwin, Shaveta Vinayak, Nancy E Davidson, Hannah M Linden

Table S1. Log-Rank test between variables including HER2 status, FES-PET and FDG-PET uptake. FDG uptake was the only significant variable.

Group	TTP events	Survival events
FES High	79	95
FES Low	78	95
FDG High	79	95
FDG Low	78	95
HER2+	26	28
HER2-	131	162

	**P-value using Log-Rank Test**		
**Separator**	**Comparison**	**Survival**	**TTP**
HER2 Status	HER2+ vs HER2-	0.087	0.323
Median	FES High vs FES Low	0.289	0.229
Median	FDG High vs FDG Low	<0.001	<0.001

Serial scan analysis

Using the same inclusion criteria, a subset of patients who underwent at least two paired FDG and FES scans had their additional scans compared to their first scans and reviewed further for variability over time. These serial scans allowed tracking FES uptake over multiple timepoints. Table S2 shows the distribution of histology, lesion type, FES and FDG uptake measures as well as the percentage change and unit change from the time of the first FES or FDG scan to subsequent scans. There were 2 patients that had their FES scans

done on two different machines (one 2 years after the first scan and the other 6 years after the first scan), but the machines were calibrated identically. Images from one patient are illustrated in Figure S1. Figure S2 depicts the average FES and FDG SUVmax uptake across each study in each of the patients with serial imaging. With few exceptions, FES avidity remained stable over time in both the HER2-positive and HER2-negative populations despite the fact that many underwent treatment during the interim.

This stability of estrogen receptor density in patients over time is notable and suggests that patients may maintain endocrine sensitivity for some time despite treatment with HER2- and ER- targeted agents, much like their ER-positive, HER2-negative counterparts. The observations, however, may be due to patient selection, as many patients included in our cohort underwent imaging as part of clinical trials that required planned endocrine therapy. Thus, clinicians referring a patient to one of these FES studies were likely acting on clinical intuition that the ER pathway may be important for that patient, so these data may not be reflective of the overall population with triple-positive tumors.

Table S2. Patients with additional scans

	Total<br>n=38 patients<br>n=91 scans<br>n=430 lesions
**# with 2 scans**<br>**# with 3 scans**<br>**# with 4 scans**	n= 24<br>n=13<br>n= 1
**Histology**	
Ductal	30
Lobular	7
Unknown	0
Other	1
**Lesion Distribution: n (%)**	
**Bone**	292 (68%)
**Soft tissue/lung**	138 (32%)
**Time between FES scan 1 and all others: mean (range)**	724 (15-3165)
**FES average SUVmax: mean (range)**	
Average FES SUVmax across all studies	3.5 (0.7-12.6)
Average FES SUVmax across all lesions	3.8 (0.3-22.8)
**Average FES change between scans across all studies: mean (range)**	
% change from 1st scan	18.7 (-76-530)
Unit change from 1st scan	0.10 (-3.1-3.9)
% change from closest scan	13.3 (-76-530)
Unit change from closest scan	-0.04 (-3.1-3.9)
**FDG average SUVmax: mean (range)**	
FDG SUVmax across all studies	4.8 (1.5-11.3)
FDG SUVmax across all lesions	5.0 (1.3-17.1)
**FDG change between scans: mean (range)**	
% change from 1st scan	25.1 (-80.7-307.9)
Unit change from 1st scan	0.34 (-7.5-8.5)
% change from closest scan	12.1 (-80.7-200.3)
Unit change from closest scan	0.10 (-7.5-5.5)

Figure S1. 70-year-old woman with ER+/PR+/HER2-positive MBC treated with endocrine therapy + trastuzumab. FDG and FES SUVmax correlates across scans as tumor progresses; the patient died of disease 10 years after baseline imaging.

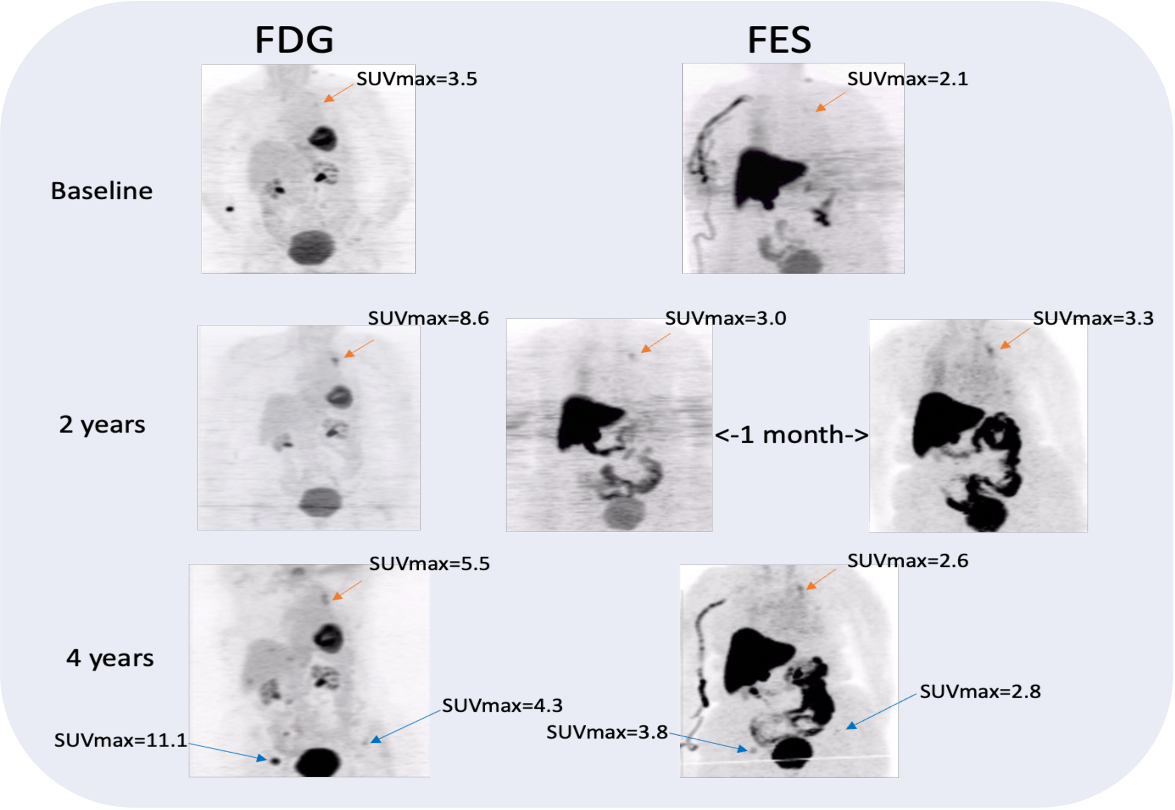

Figure S2. Comparison of average FES SUVmax and FDG SUVmax across all studies in 38 patients with serial imaging.

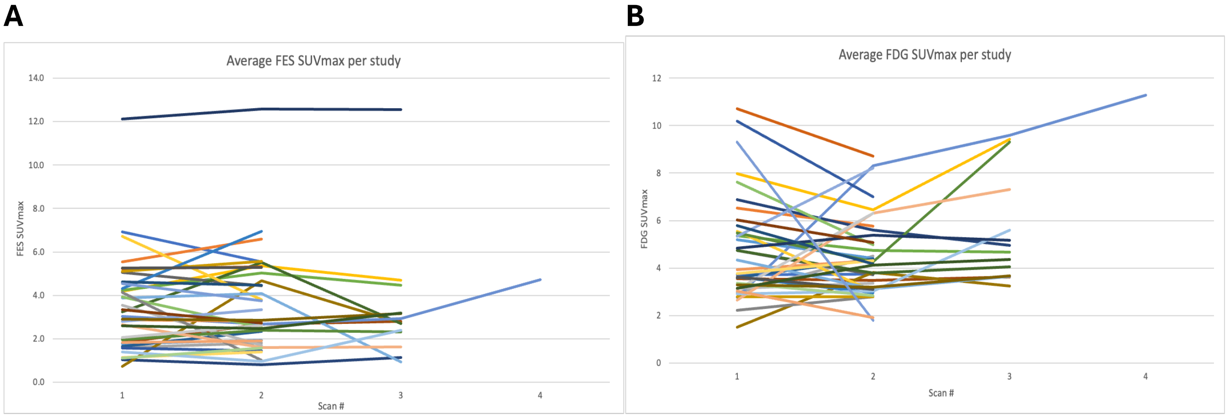

Supplement: Supplementary file 1 — Supplementary Material 1 [file 13058_2025_1975_MOESM1_ESM.pdf]
